# Toward Brain Na_V_1.8 Imaging with [^11^C]Suzetrigine

**DOI:** 10.3390/ph18121816

**Published:** 2025-11-28

**Authors:** Ramya Tokala, Torben D. Pearson, Braeden A. Mair, Sarah Bricault, Rachel Wallace, Hsiao-Ying Wey, Jacob M. Hooker, So Jeong Lee

**Affiliations:** 1 Athinoula A. Martinos Center for Biomedical Imaging, Department of Radiology, Massachusetts General Hospital, Harvard Medical School, Charlestown, MA 02129, USA; 2 McLean Imaging Center, McLean Hospital, Belmont, MA 02478, USA

**Keywords:** Na_V_1.8, positron emission tomography (PET), pain imaging, radiotracer development, central nervous system (CNS)

## Abstract

**Background/Objective:** Acute and chronic pain affect millions of individuals, yet there are currently no molecular imaging tools to directly assess pain-related mechanisms in the central nervous system (CNS). The voltage-gated sodium channel Na_V_1.8 plays a pivotal role in neuropathic pain by increasing the excitability of nociceptive neurons following nerve injury or inflammation. In this work, we aimed to develop a novel positron emission tomography (PET) imaging probe for Na_V_1.8 to facilitate noninvasive quantification of this target in the CNS and thereby advance our understanding of pain neurobiology. **Methods:** We selected the compound suzetrigine, a U.S. FDA-approved, highly selective non-opioid Na_V_1.8 inhibitor, as the first candidate for a Na_V_1.8-targeted PET tracer. The compound was first assessed using in silico docking and CNS multiparameter optimization (MPO) analysis to evaluate target binding and predicted brain penetrability. Radiolabeling was accomplished by *O*-methylation with [^11^C]methyl iodide to yield [^11^C]suzetrigine without structural modification. The tracer was then evaluated using in vitro binding assays, including autoradiography and saturation binding on rat brain tissues, to determine binding parameters (K_D_, B_max_), and using in vivo PET imaging in rats to assess brain uptake, time–activity curves (TACs), and tracer behavior under baseline and pretreatment conditions. Pretreatment was performed with unlabeled suzetrigine, the P-glycoprotein (P-gp) inhibitor verapamil, and the heterologous Na_V_1.8 inhibitor A-803467. **Results:** In silico docking demonstrated favorable binding of suzetrigine to the Na_V_1.8 active site, and the calculated CNS MPO score (>3.5) suggested adequate brain penetration. Radiochemical synthesis of [^11^C]suzetrigine via *O*-methylation yielded a high decay-corrected radiochemical yield (19.2 ± 2.7%, *n* = 3), excellent purity (>98%, *n* = 3), and moderate molar activity (62.9 ± 51.8 MBq/nmol, *n* = 3). Autoradiography on rat brain tissue confirmed saturable and selective binding of [^11^C]suzetrigine to Na_V_1.8. Saturation binding assays revealed a B_max_ = 93 fmol/mg and a K_D_ = 0.1 nM, supporting the imageability of Na_V_1.8 in the brain using this tracer. In vivo PET imaging in rats demonstrated rapid and sufficient brain uptake but revealed unexpected tracer behavior: signal intensity markedly increased following pretreatment with either unlabeled suzetrigine or the P-gp inhibitor verapamil, and showed a slight increase after pretreatment with the heterologous Na_V_1.8 inhibitor A-803467. Detailed analysis of PET images, TACs, and normalized area-under-curve (AUC) values indicated that these atypical uptake patterns were primarily attributable to P-gp-mediated effects, although additional factors may also contribute. **Conclusions:** [^11^C]Suzetrigine exhibits high affinity, good brain uptake, and selective target engagement in vitro, supporting its potential as a first-in-class Na_V_1.8-PET tracer. However, in vivo performance is confounded by P-gp-mediated efflux and possibly other mechanisms that limit accurate quantification of Na_V_1.8 in the living brain. These findings underscore the critical role of efflux transporters in CNS radiotracer development and highlight the need for design strategies that mitigate P-gp interaction when targeting ion channels in the brain. Future studies will include imaging under constant P-gp inhibition, arterial blood sampling for radiometabolite analysis and full kinetic modeling, and evaluation in non-human primates to assess translational feasibility.

## 1. Introduction

Pain, whether acute or chronic, remains one of the most pervasive and challenging medical conditions worldwide, imposing a profound burden on quality of life and healthcare systems [1,2]. Acute pain, typically defined as lasting less than three months, commonly arises from injury, surgery, or inflammation and is generally reversible with appropriate treatment [3,4]. In the United States alone, more than 80 million individuals are prescribed medications each year for acute pain, underscoring its immense clinical and socioeconomic impact [5]. Despite substantial progress in analgesic development, a significant portion of patients experience inadequate relief or transition to chronic pain, a complex condition characterized by sustained sensitization of nociceptive pathways and maladaptive neuroplasticity. This transition remains difficult to predict or prevent due to the lack of reliable and validated biomarkers capable of monitoring pain-related neurobiology or identifying individuals at risk for chronification [6,7]. Although positron emission tomography (PET) imaging using translocator protein (TSPO) ligands has been explored to assess neuroinflammation in pain disorders, these approaches are indirect and not specific to nociceptive processing [6,7]. Advances in PET now offer a unique opportunity to bridge this gap by visualizing pain-related molecular targets in vivo and linking neurochemical mechanisms to clinical pain states [7].

In response to these unmet needs, extensive research has focused on identifying non-opioid molecular targets that underlie pain signaling. One of the most promising families of such targets is voltage-gated sodium (Na_V_) channels, which play essential roles in the initiation and propagation of action potentials in excitable cells [8,9,10,11]. Among the nine known Na_V_ subtypes (Na_V_1.1 to Na_V_1.9), Na_V_1.7, Na_V_1.8, and Na_V_1.9 are predominantly expressed in peripheral sensory neurons and are critically involved in nociceptive transmission [12]. Na_V_1.8, in particular, is selectively expressed in nociceptive neurons of the dorsal root ganglia (DRG) and contributes to hyperexcitability and pain hypersensitivity following nerve injury or inflammation [13]. Its expression and functional contribution increase across multiple neuropathic and inflammatory pain states. In rodent models, sciatic nerve injury produces marked upregulation of Na_V_1.8 mRNA and immunoreactivity in affected axons, while cancer-induced bone pain enhances Na_V_1.8 membrane localization in DRG neurons [14,15,16]. Likewise, focal accumulation of Na_V_1.8 protein has been observed in human peripheral nerves at sites of neuropathic injury [17,18,19]. These convergent findings highlight Na_V_1.8 as a biologically validated, peripherally enriched pain transducer and support its appeal as a therapeutic target for non-opioid analgesics designed to modulate peripheral nociceptive signaling without central opioid-related side effects [20,21].

**Figure 1:**
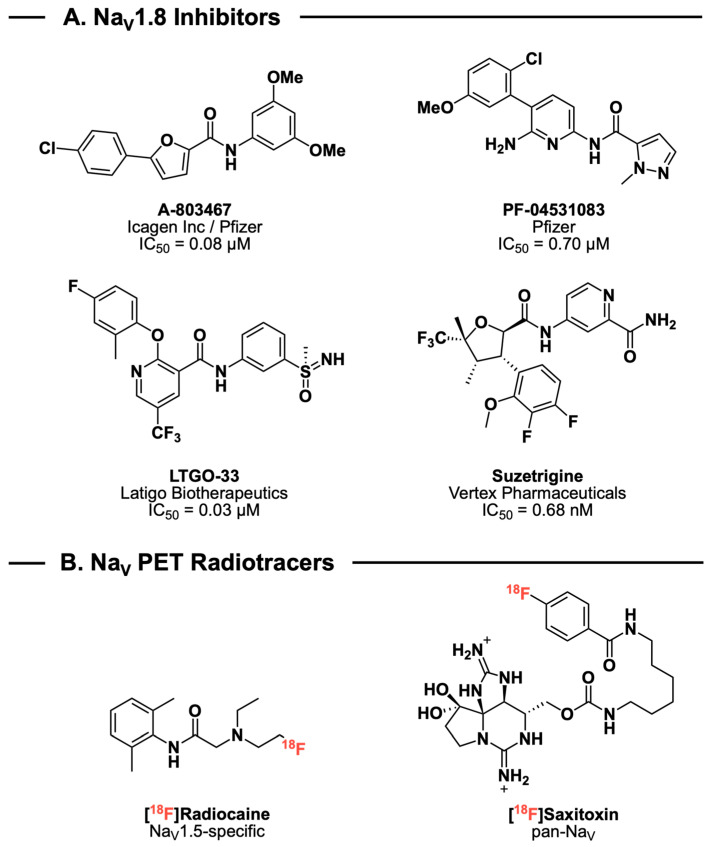
Development of Na_V_1.8 inhibitors and Na_V_-targeted PET Radiotracers: (**A**) Representative Na_V_1.8 inhibitors that have been investigated for clinical use. (**B**) Selected examples of promising ^18^F-labeled PET radiotracers developed for imaging Na_V_ channel expression in vivo.

Over the past two decades, intensive medicinal chemistry and pharmacological efforts have yielded potent and selective Na_V_1.8 inhibitors (Figure 1A). Among them, suzetrigine (VX-548, Journavx^®^; Vertex Pharmaceuticals, Boston, MA, USA) has emerged as a clinically validated, Na_V_1.8 inhibitor (IC_50_ = 0.68 ± 0.16 nM) with favorable pharmacokinetic properties and a strong safety profile [21,22,23,24,25,26,27,28]. In 2025, suzetrigine received U.S. FDA approval as the first non-opioid analgesic in a new therapeutic class targeting Na_V_1.8 for the treatment of peripheral neuropathic pain [5]. This milestone established Na_V_1.8 as a clinically actionable target and signaled a paradigm shift away from opioid-based pain therapy. However, despite its well-defined peripheral role, the expression and function of Na_V_1.8 within the central nervous system (CNS), particularly in regions such as the thalamus, amygdala, and limbic cortex involved in pain perception, affect, and chronification, remain poorly characterized [18]. The limited understanding of its CNS distribution and regulation complicates translational research and hinders the development of imaging biomarkers that could quantify its engagement in vivo.

PET provides a powerful, quantitative approach to measure molecular targets in the living brain with high sensitivity and specificity. By enabling direct assessment of target distribution, density, and drug occupancy, PET bridges molecular pharmacology and systems-level neurobiology. In the pain field, however, PET applications have largely focused on opioid receptors and neuroinflammatory markers [7]. The development of subtype-selective PET radiotracers for Na_V_ channels remains in its infancy, with most prior work centering on Na_V_1.5 or non-selective inhibitors lacking adequate subtype specificity or CNS penetrance (Figure 1B) [29,30]. A Na_V_1.8-selective PET tracer would represent a major advancement, allowing for noninvasive visualization of its distribution and potential contribution to central pain mechanisms.

While peripheral Na_V_1.8 is well characterized, its expression and functional role in the CNS remain poorly defined, limiting translational studies across species, including imaging. Emerging human transcriptomic datasets report little to no detectable SCN10A (Na_V_1.8) mRNA in the brain [27], yet this does not necessarily preclude low-level or region-specific protein expression. Discrepancies between transcript and protein abundance are well recognized, particularly for ion channels subject to complex post-transcriptional regulation (e.g., alternative splicing, epigenetic control) [31,32,33,34]. Although protein-level validation of Na_V_1.8 in the brain through Western blot or immunohistochemistry would be definitive, such analyses are currently limited by the absence of antibodies validated for Na_V_1.8 detection in CNS tissue. In this context, an in vivo imaging probe offers a complementary approach to determine whether functional Na_V_1.8 protein is present in the brain and to evaluate its potential contribution to central pain modulation.

In this study, we evaluated suzetrigine, a clinically validated and highly selective Na_V_1.8 inhibitor, as a candidate molecular probe for Na_V_1.8-PET imaging. Its favorable physicochemical characteristics, including adequate molecular weight, lipophilicity, and CNS multi-parameter optimization (MPO) score of ≥3.5, suggest potential for brain penetration [35,36]. We retained its native pharmacophore and radiolabeled suzetrigine with carbon-11 at the *O*-methyl position to generate [^11^C]suzetrigine. Using a multimodal approach that integrated in silico docking, in vitro autoradiography, and in vivo dynamic PET imaging in healthy rats, we characterized the tracer’s pharmacokinetics, binding selectivity and affinity, target density, brain uptake, and interaction with efflux transporters. This work represents the first attempt to image Na_V_1.8 in the living brain using PET, providing new insights into the quantitative imaging of pain mechanisms and informing the design of next-generation Na_V_-channel imaging agents.

## 2. Results and Discussion

### 2.1. In Silico Evaluation of Suzetrigine as a Na_V_1.8 Targeted CNS PET Tracer Candidate

#### 2.1.1. Molecular Docking of Suzetrigine in the Human Na_V_1.8 Binding Pocket

**Figure 2:**
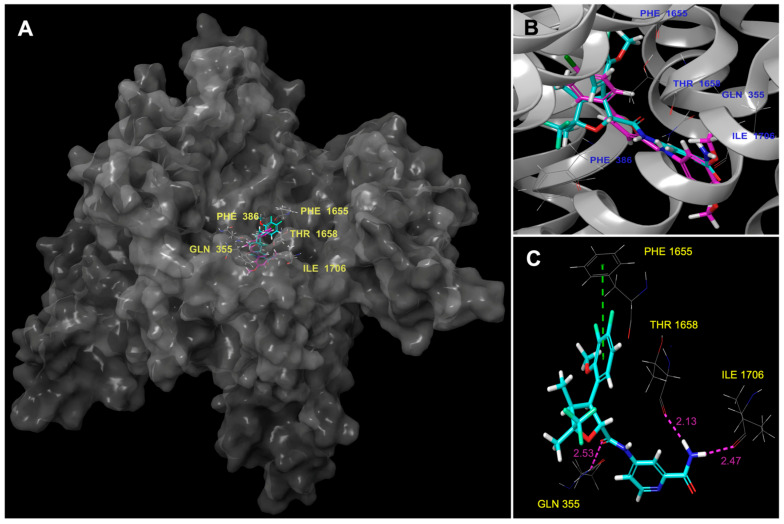
Molecular docking of suzetrigine within the Na_V_1.8 binding site defined by the crystallographic ligand A-803467 (PDB ID: 7WE4): (**A**) Superimposed surface view of predicted binding pose of suzetrigine (cyan) overlaid with crystallographic A-803467 (magenta) within the active pocket of human Na_V_1.8 (7WE4). Key interacting amino acid residues lining the pocket are shown in yellow. (**B**) Close-up view of the superimposed ligand poses highlighting shared interactions between suzetrigine and A-803467 with key binding-site residues (blue), including hydrogen bonding and π–π stacking contacts. (**C**) Best-scoring binding pose of suzetrigine showing hydrogen bond interactions with Gln355, Thr1658, and Ile1706 and π–π stacking with Phe1655. Distances (magenta) indicate atom-atom separations involved in hydrogen bond interactions.

To determine whether structural modification would be required to optimize Na_V_1.8 affinity for PET tracer development, where radiolabeled compounds are typically administered at picomolar concentrations, we performed molecular docking using the established human Na_V_1.8 cryo-EM structure co-crystalized with A-803467 (PDB ID: 7WE4) in Schrödinger 2024-4 (Figure 2; details in Section 3.1. Molecular docking) [37,38]. Suzetrigine was docked into the same binding pocket occupied by A-803467 in the co-crystal structure (Figure 2A), and the two ligands assumed clearly overlapping poses (Figure 2B). The predicted interaction profile of suzetrigine closely paralleled that of A-803467, engaging the pocket through a combination of hydrogen bonding, π–π interactions, and hydrophobic contacts (Figure 2C and Figure S4). For example, the bridged carbonyl oxygen (C=O) of suzetrigine formed a hydrogen bond with the amide N–H of Gln355 (2.5 Å), mirroring the same hydrogen bond observed for A-803467. A π–π interaction was identified between the difluoro-substituted phenyl ring of suzetrigine and Phe1655, similar to the aromatic interaction between A-803467 and Phe386. Several hydrophobic contacts were shared with residues Ile381, Phe382, Phe386, Phe1710, Met1713, and Met1716. These interaction features are consistent with the reported binding mode of A-803467 and support the experimentally observed high potency and selectivity of suzetrigine for Na_V_1.8 [27,28]. The overlapping interaction pattern further indicates that suzetrigine and A-803467 occupy a common active site, supporting the rationale for using A-803467 as a heterologous competitor in the in vitro displacement assays (see Section 2.3.1. Competition binding).

#### 2.1.2. In Silico Prediction of Brain Uptake

Molecular descriptor analysis was performed to evaluate the physicochemical suitability of suzetrigine for CNS imaging [35,36,39]. The calculated descriptors included (i) ClogP, (ii) ClogD, (iii) molecular weight (MW), (iv) topological polar surface area (TPSA), (v) number of hydrogen-bond donors (HBDs), and (vi) pK_a_ of the most basic center. The resulting CNS multiparameter optimization (MPO) score for suzetrigine was 3.51 (Table S2), positioning suzetrigine near the empirical threshold for brain penetration. Among key determinants for of permeability—such as ClogP, ClogD, and TPSA—ClogP and ClogD values (within the 2–4 range) fell within the favorable window for PET tracers, whereas the TPSA slightly exceeded the optimal threshold (<90 Å^2^). Other descriptors, such as MW, HBD, and pK_a_, contributed minimally to distinguishing successful from failed CNS tracers [36]. Collectively, these findings indicate that suzetrigine possesses physicochemical properties at the boundary of CNS penetrability yet remains a plausible scaffold for Na_V_1.8-targeted PET tracer development.

In combination with its clinical validation, subnanomolar potency, and structurally simple *O*-methyl handle suitable for direct [^11^C]methylation without disrupting the pharmacophore, suzetrigine was prioritized for subsequent radiochemical synthesis and experimental evaluation.

### 2.2. Radiosynthesis of [^11^C]Suzetrigine

**Scheme 1:**
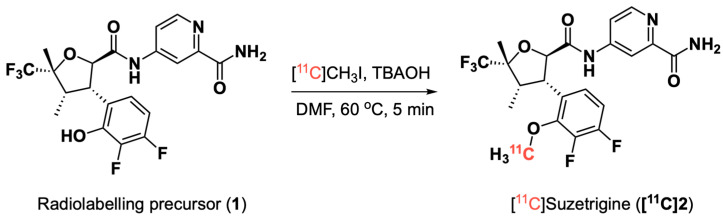
Radiosynthesis of [^11^C]suzetrigine. *O*-^11^C-methylation of the desmethyl precursor (**1**) using [^11^C]CH_3_I, and TBAOH in DMF yielded [^11^C]suzetrigine ([^11^C]**2**).

Given that suzetrigine is a pharmacologically and biologically well-characterized Na_V_1.8 inhibitor, its native pharmacophore was retained for radiolabeling with carbon-11. The radiosynthetic method is outlined in Scheme 1. The desmethyl precursor of suzetrigine (**1**) was methylated using [^11^C]methyl iodide ([^11^C]CH_3_I) in the presence of TBAOH at 60 °C to afford the *O*-^11^C-methylated product, [^11^C]suzetrigine ([^11^C]**2**). The crude reaction mixture was purified by reverse-phase semi-preparative HPLC and subsequently reformulated in 10% ethanol/90% saline to yield the final injectable product in a decay-corrected radiochemical yield of 19.2 ± 2.7% (non-decay-corrected yield: 5.7 ± 0.7%) (*n* = 3). The total activity of [^11^C]**2** at the end of synthesis (EOS) was 569.8 ± 140.6 MBq (*n* = 3), obtained from 9472 ± 1380.1 MBq of [^11^C]CH_3_I produced by the cyclotron. The radiochemical purity was ≥98% (*n* = 3), and the molar activity was 62.9 ± 51.8 MBq/nmol (non-decay corrected) at EOS (*n* = 3) after an overall synthesis time of approximately 40 min from the end of cyclotron bombardment. The chemical identity of [^11^C]**2** was confirmed by analytical radio-HPLC through co-elution with the nonradioactive suzetrigine reference standard (see Table S1, and Figures S1–S3 in Supplementary Materials).

### 2.3. In Vitro Autoradiography Study

#### 2.3.1. Competition Binding

**Figure 3:**
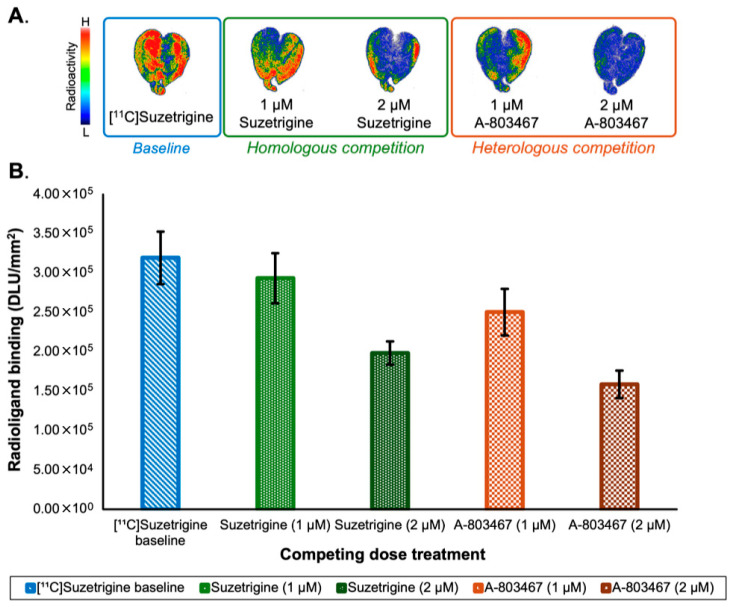
Competitive blocking of [^11^C]suzetrigine binding in rat brain tissue assessed by in vitro autoradiography: (**A**) Representative autoradiography image from the competition study using rat brain sections in transverse orientation. (**B**) Quantitative comparison of [^11^C]suzetrigine binding under baseline conditions and during co-incubation with unlabeled suzetrigine or the heterologous Na_V_1.8 inhibitor A-803467 at 1 and 2 µM concentrations. Baseline sections were incubated with [^11^C]suzetrigine alone, whereas blocking sections were co-incubated with the indicated concentrations of suzetrigine or A-803467. Data are presented as mean ± standard deviation (SD). Quantification was performed from unbiasedly selected image-based regions of interest (ROIs), with *n* = 6 for baseline, suzetrigine (1 µM and 2 µM), and A-803467 (2 µM), and *n* = 3 for A-803467 (1 µM). Each ROI represented an independent measurement of local binding intensity (see Section 3.3. In vitro autoradiography, Competition binding assays).

In vitro competition autoradiography demonstrated that [^11^C]suzetrigine binds to a displaceable, target-related site in rat brain tissue (Figure 3). A-803467 was included as a heterologous competitor because our docking analysis indicated that it occupies the same Na_V_1.8 binding pocket as suzetrigine (Figure 2), thereby providing an orthogonal confirmation of target-mediated displacement. Although A-803467 is substantially less potent than suzetrigine (approximately 120-fold, Figure 1), its well-characterized binding mode and selectivity made it an appropriate complementary comparator in these feasibility assays. Under baseline conditions, [^11^C]suzetrigine displayed a heterogeneous distribution across brain regions, whereas pretreatment with either the unlabeled homologous compound (suzetrigine) or the heterologous Na_V_1.8 inhibitor A-803467 markedly reduced the autoradiographic signal (Figure 3A). At 2 μM concentration, suzetrigine and A-803467 decreased [^11^C]suzetrigine binding by approximately 38% and 50%, respectively, while 1 μM of each compound produced only around 8% and 21% reductions in signal (Figure 3B). The diminished blocking at the lower concentrations supports a concentration-dependent, saturable binding relationship to a finite population of sites. Together, these results indicate that a substantial proportion of the observed signal arises from specific, target-related binding rather than nonspecific membrane interactions. The incomplete displacement at the highest concentrations suggests the presence of a non-displaceable component, which may reflect nonspecific binding, incomplete equilibrium, or partial overlap of binding sites. Such partial displacement is commonly observed for ion-channel radioligands due to the presence of membrane-associated nonspecific binding components [40,41]. Nevertheless, the dose-dependent homologous competition and reproducible reduction by a structurally distinct inhibitor collectively demonstrate saturability and selectivity of [^11^C]suzetrigine binding to Na_V_1.8. Given that these assays were designed primarily to verify target engagement before proceeding to in vivo PET evaluation, the observed displacement profile provided sufficient justification to advance to the next stage of tracer characterization.

#### 2.3.2. Saturation Binding

**Figure 4:**
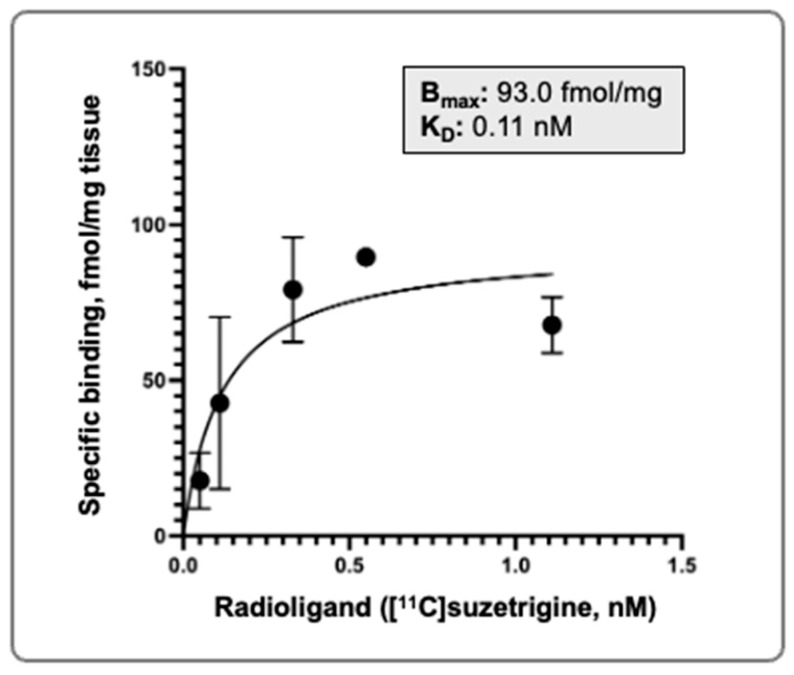
Saturation binding of [^11^C]suzetrigine determined by in vitro autoradiography in female Sprague Dawley rat brain sections. Saturation binding curve of [^11^C]suzetrigine demonstrating specific, high-affinity binding with a K_D_ of 0.11 nM and a B_max_ of 93.0 fmol/mg tissue. The *x*-axis represents the concentration of [^11^C]suzetrigine (0–1.5 nM), and the *y*-axis denotes specific binding (fmol/mg tissue). Data are expressed as mean ± SD, and K_D_ and B_max_ values were obtained by nonlinear regression analysis in GraphPad Prism v10.4.1 (see Section 3.3. In vitro autoradiography, Saturation binding assays).

To further characterize [^11^C]suzetrigine binding parameters, saturation autoradiography was performed using the unlabeled homologous compound to define affinity (K_D_) and maximal binding capacity (target protein density, B_max_). Incremental concentrations of [^11^C]suzetrigine (0.05, 0.1, 0.3, 0.5, and 1.1 nM) were incubated with adjacent rat brain sections in the presence or absence of a fixed blocking dose (70 nM; approximately 100-fold higher than the reported IC_50_ of suzetrigine, 0.68 nM [27,28]. Total and nonspecific binding were measured, and specific binding was derived as their difference (see Section 3.3. In vitro autoradiography, Saturation binding assays and Figure S5). The saturation curve presented in Figure 4 demonstrates the specific binding. Nonlinear regression analysis yielded an estimated B_max_ of 93.0 fmol/mg tissue and a K_D_ of 0.1 nM, indicating high-affinity, saturable binding consistent with the reported in vitro potency of suzetrigine. The measured K_D_ (0.1 nM) is within an order of magnitude of the reported IC_50_ (0.68 nM), consistent with expected radioligand behavior. These values suggest that Na_V_1.8 binding sites are present in rat brain tissue at densities sufficient to permit quantitative PET imaging.

Collectively, the competition and saturation autoradiography results demonstrate that [^11^C]suzetrigine binds selectively to a finite number of high-affinity, displaceable sites consistent with Na_V_1.8. The combination of homologous and heterologous blocking, coupled with quantitative binding parameters, supports specific and saturable target engagement in vitro. These autoradiography findings align with prior reports that ion-channel radioligands often display partial displaceability due to a mix of specific and membrane-associated binding, yet still provide sufficient target contrast for translational PET evaluation. Based on these findings, subsequent in vivo PET imaging studies were conducted to evaluate tracer kinetics, brain uptake, and selectivity under physiological conditions.

### 2.4. In Vivo PET Imaging and Assessment of Brain Uptake

#### 2.4.1. Baseline Tracer Kinetics and Image-Based Regional Distribution

**Figure 5:**
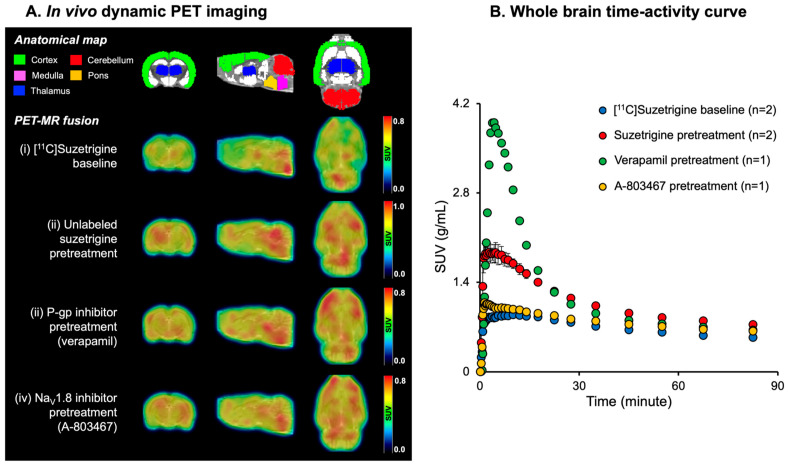
In vivo PET imaging of [^11^C]suzetrigine in the rat brain: (**A**) PET images reconstructed with CT-based attenuation correction and registered to an MR atlas space. PET-MR fusion summed brain images (60–90 min) show representative tracer distribution under the following conditions: (i) baseline (*n* = 2); (ii) pretreatment with unlabeled suzetrigine (1 mg/kg, i.v.) (*n* = 2); (iii) pretreatment with the P-gp inhibitor verapamil (1 mg/kg, i.v.) (*n* = 1); (iv) pretreatment with the Na_V_1.8 inhibitor A-803467 (1 mg/kg, i.v.) (*n* = 1). (**B**) Corresponding time–activity curves (TACs) illustrating dynamic brain uptake of [^11^C]suzetrigine for conditions (i)–(iv).

Given suzetrigine’s clinical validation as an FDA-approved analgesic, its subnanomolar affinity for Na_V_1.8, acceptable predicted CNS penetration, preserved pharmacophore after radiolabeling, and demonstrated selectivity and saturability at the Na_V_1.8 binding site in silico and in vitro, we prioritized baseline in vivo PET imaging as the most direct and informative evaluation of brain entry and whole-brain pharmacokinetics for [^11^C]suzetrigine. Accordingly, dynamic in vivo PET imaging was performed in healthy female Sprague Dawley rats (6–8 weeks old) to assess the tracer’s brain penetrability and in vivo pharmacokinetic behavior. A sterile formulation (10% ethanol in saline, 25.9–33.3 MBq) was administered intravenously, followed by a 90-minute dynamic PET acquisition. Figure 5A shows representative PET-MR fusion images illustrating whole-brain distribution (see Section 3.4. In vivo PET imaging, Rodent PET imaging analysis), and Figure 5B depicts the corresponding time–activity curves (TACs) expressed as standardized uptake values (SUVs). At baseline, [^11^C]suzetrigine exhibited rapid initial brain entry, reaching a peak SUV of approximately 0.8 g/mL within 1–5 min post-injection. Uptake briefly plateaued until around 15 min and then slowly declined throughout the 90-minute scan. Regional analysis revealed the highest activity in the thalamus, followed by pons, medulla, and cerebellum (Figure 5A), consistent with moderate passive diffusion but limited net retention. These findings indicate that [^11^C]suzetrigine is capable of crossing the blood–brain barrier (BBB) but that its baseline uptake is relatively low and washout slow, suggesting possible involvement of active efflux processes or mixed nonspecific binding.

#### 2.4.2. Effect of Suzetrigine Pretreatment

To explore how the unlabeled compound influences tracer distribution, animals were administered suzetrigine intravenously 10 min before tracer injection. Such pretreatment is routinely used in exploratory radiotracer studies to probe target engagement qualitatively. Surprisingly, rather than decreasing uptake, suzetrigine pretreatment produced a 1.5–2-fold increase in whole-brain signal, with a peak SUV of about 1.8 g/mL that gradually declined over time (Figure 5B). This unexpected elevation suggests altered tracer delivery rather than specific target blockade. Possible explanations include (1) an increase in the plasma-free fraction due to peripheral target occupancy by unlabeled suzetrigine, (2) modulation of BBB efflux transporters (e.g., P-glycoprotein, P-gp), or (3) additional nonspecific or off-target interactions in the CNS. Given the logistical challenges of blood sampling and metabolite correction in rodents, direct quantification of these effects was not feasible; therefore, subsequent PET studies focused on evaluating potential efflux contributions.

#### 2.4.3. Effect of P-gp Inhibition on Tracer Uptake

To determine whether [^11^C]suzetrigine interacts with efflux transporters at the BBB, animals were pretreated with verapamil, a prototypical P-gp inhibitor known to enhance brain delivery of P-gp substrates [42,43,44]. P-gp (ABCB1) is the dominant efflux transporter limiting brain exposure of many xenobiotics across species [45,46]. Consistent with a P-gp-limited transport profile, verapamil pretreatment (1 mg/kg, i.v.) markedly increased brain uptake of [^11^C]suzetrigine, yielding a 4-fold higher peak SUV (approximately 4 g/mL) within 5 min post-injection, followed by rapid clearance within 10 min (Figure 5B). This pattern aligns with reports that P-gp inhibition transiently elevates brain levels of P-gp substrates before accelerated washout once equilibrium is re-established. Complementary in vitro autoradiography confirmed that verapamil co-incubation (1 or 2 μM) produced <18% change in binding intensity (Figure S6), supporting the interpretation that the in vivo effect arises from BBB transport modulation rather than altered affinity or tissue binding. Collectively, these data indicate that [^11^C]suzetrigine likely interacts with P-gp at the BBB, limiting its baseline penetration and complicating quantitative assessment of Na_V_1.8-specific binding in vivo.

#### 2.4.4. Pretreatment with Heterologous Na_V_1.8 Inhibitor

A separate PET experiment examined the effect of A-803467, a selective Na_V_1.8 inhibitor predicted from docking and in vitro data to bind the same active site on Na_V_1.8 as suzetrigine. Contrary to expectations of signal reduction, brain uptake following A-803467 pretreatment remained comparable to baseline, with only minor fluctuations in TACs (Figure 5B). This result could have several explanations. First, if A-803467 shows limited brain penetration, it would predominantly occupy peripheral Na_V_1.8 sites (e.g., in dorsal root ganglia), thereby increasing the plasma free fraction of [^11^C]suzetrigine and transiently enhancing its apparent brain uptake [40]. Alternatively, A-803467 could itself interact with P-gp, indirectly modulating tracer delivery. Together, these findings suggest that the observed signal changes primarily reflect peripheral or transporter-mediated effects rather than displacement of specific Na_V_1.8 binding in the brain.

#### 2.4.5. Regional Uptake Comparison

**Figure 6:**
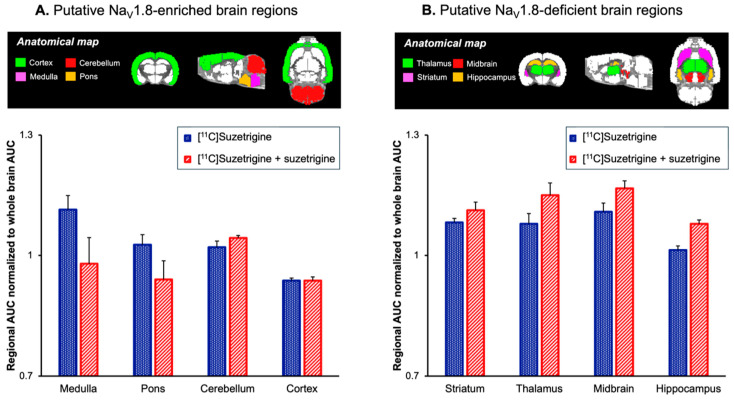
Regional analysis of normalized [^11^C]suzetrigine uptake under baseline and homologous pretreatment conditions. To account for the influence of P-gp-mediated efflux, regional brain uptake was normalized to the corresponding whole-brain area under the curve (AUC), where a normalized value of 1.0 indicates uptake equal to the whole-brain mean: (**A**) Normalized AUC values derived from time–activity curves (TACs) in putative Na_V_1.8-enriched regions—cortex, medulla, pons, and cerebellum (*n* = 2 per condition). (**B**) Normalized AUC values in putative Na_V_1.8-deficient regions—thalamus, striatum, midbrain, and hippocampus (*n* = 2 per condition). Regional classifications were based on transcriptomic reference data [19,47,48].

To further examine potential target-related regional trends, brain uptake (AUC_0–90min_) was analyzed by region, using the whole brain as a pseudo-reference to account for P-gp-mediated variability under homologous pretreatment conditions (Figure 6 and Figure S7). Because in vitro autoradiography confirmed measurable Na_V_1.8 binding sites in rat brain tissue (B_max_ = 93 fmol/mg tissue; see Section 2.3.2. Saturation binding), transcriptomic data from the Allen Mouse Brain Atlas were used only as a tentative reference for regional expression. According to the Atlas, SCN10A (encoding Na_V_1.8) transcripts are most abundant in the cortex, pons, medulla, and cerebellum [47]. Based on transcriptomic surveys [19,48], brain regions such as the thalamus, striatum, midbrain, and hippocampus show very low or undetectable SCN10A expression, suggesting they may be considered as putative Na_V_1.8-deficient regions. It should be noted, however, that mRNA abundance does not necessarily correspond to protein density, particularly for ion channels subject to complex post-transcriptional regulation [31,32,33,34], so regional transcriptomic patterns were interpreted with caution and compared qualitatively against experimentally derived in vitro and in vivo data. After normalization, modest reductions (approximately 25–30%) in normalized uptake were observed in the brainstem under suzetrigine pretreatment, whereas cerebellum and cortex showed no appreciable change (Figure 6A). Notably, the thalamus, where Na_V_1.8 transcripts are minimal, displayed persistently high uptake under both baseline and homologous pretreatment conditions (Figure 6B), suggesting that much of the observed signal may reflect nonspecific or transport-related components rather than Na_V_1.8-specific binding. There was no signal reduction observed in the other regions classified as putative Na_V_1.8-deficient brain regions. No consistent reduction was detected in other regions known to be Na_V_1.8-deficient. Due to the lack of a validated reference region for Na_V_1.8, full quantitative kinetic modeling and binding potential estimation were not performed, and analyses were therefore limited to semiquantitative interpretation based on SUV and normalized AUC measures. Thus, these regional analyses provide qualitative insight into tracer distribution patterns rather than definitive measures of specific binding.

#### 2.4.6. Interpretation and Limitations

Overall, in vivo PET imaging demonstrated that [^11^C]suzetrigine can enter the brain but that its signal is dominated by factors unrelated to specific Na_V_1.8 binding. The pronounced increase in uptake after verapamil or suzetrigine pretreatment strongly implicates P-gp-mediated efflux as a major determinant of its brain pharmacokinetics. Without arterial sampling or a validated reference region, quantitative modeling could not be performed; therefore, these results should be viewed as proof-of-concept findings illustrating key translational barriers. Future optimization of this scaffold should focus on reducing P-gp interaction and enhancing BBB permeability to enable reliable quantification of Na_V_1.8 in vivo.

## 3. Materials and Methods

### 3.1. Molecular Docking

The crystal structure of the human Na_V_1.8 was obtained from the Protein Data Bank (PDB ID: 7WE4) [49]. Protein preparation was performed using the Protein Preparation Wizard in Schrödinger 2024-4. Missing side chains and hydrogen atoms were added, bond orders were assigned, and all crystallographic water molecules located more than 5 Å from the active site were removed. A three-dimensional, precomputed ligand-protein energy grid was generated by centering on the co-crystallized ligand binding pocket, defining a cubic grid box of 10 × 10 × 10 Å. The ligand suzetrigine was sketched in 2D, converted to 3D, and energy-minimized using the LigPrep module in Schrödinger 2024-4 to generate low-energy conformers. The prepared ligand conformers were docked into the active site using the Induced Fit Docking (IFD) workflow in Glide (Schrödinger 2024-4). The resulting docking poses were ranked according to GlideScore and Visual inspection of key interactions, and the top-scoring pose was selected for subsequent analysis.

### 3.2. Radiochemistry

[^11^C]Methyl iodide ([^11^C]CH_3_I) was trapped in a reaction vial preloaded with 2 mg of desmethyl precursor (**1**) and 10 μL of tetra butyl ammonium hydroxide (TBAOH, 1.0 M in methanol) dissolved in 400 µL of anhydrous N,N-dimethyl formamide (DMF). The reaction mixture was stirred at 60 °C for 5 min and subsequently quenched with 1.5 mL of water. The crude product was purified by semi-preparative HPLC using a Phenomenex Gemini-NX-C18-110Å column (250 mm × 10 mm, 5 μm) (Phenomenex, Torrance, CA, USA) and a mobile phase of 0.1% trifluoroacetic acid (TFA) in water/acetonitrile (50:50, *v*/*v*) at a flow rate of 4.0 mL/min. The desired product fraction was collected, diluted with 30 mL of water, and passed through a pre-conditioned C-18 light solid-phase extraction (SPE) cartridge (conditioned sequentially with 10 mL ethanol (EtOH) and 20 mL sterile water for irrigation, left wet). The cartridge was rinsed with 10 mL of sterile water, and the final product, [^11^C]suzetrigine ([^11^C]**2**), was eluted with 0.5 mL EtOH followed by 4.5 mL of saline (0.9%). The formulated radiotracer was used directly for in vitro autoradiography and in vivo PET imaging studies. The radiochemical and chemical purity were assessed by analytical HPLC using a Waters XBridge BEH-C18-130 Å column (4.6 mm × 150 mm, 5 μm) (Waters Corp., Milford, MA, USA) with a mobile phase of 0.1% TFA in water/acetonitrile (55:45, *v*/*v*) at a flow rate of 2 mL/min.

### 3.3. In Vitro Autoradiography

Competition binding assays: Autoradiography experiments were performed using 50 mM Tris-HCl buffer (pH 7.4), prepared by diluting 50 mL of 1 M Tris-HCl stock solution to a final volume of 1 L with distilled water. Transversal rat brain sections (female Sprague Dawley rats, 4–6 weeks old; 20 μm thickness) were pre-incubated in 100 mL of buffer for 30 min at room temperature. The buffer was then discarded, and the slides were gently blotted to remove excess moisture. Fresh buffer (100 mL) was added to new incubation boxes designated for baseline or blocking conditions. For blocking, unlabeled suzetrigine or A-803467 (1 mg dissolved in DMSO (dimethyl sulfoxide) and diluted with buffer to a final concentration of 1 μM or 2 μM) was added. In both conditions, 3.7–5.6 MBq of [^11^C]suzetrigine was introduced into the buffer (total volume = 100 mL), and sections were incubated for 10 min at room temperature. After incubation, slides were blotted and washed twice for 5 min each with fresh buffer, then air-dried carefully to prevent curling. Dried sections were exposed to phosphor imaging plates (BAS-MS2025, GE Healthcare, Piscataway, NJ, USA) under desiccation. After exposure, the plates were scanned using an image plate reader, and autoradiographic signal intensities were quantified with OptiQuant v5.0 (PerkinElmer, Waltham, MA, USA). Regions of interest (ROIs) were manually delineated based on observed visual data, and results were expressed as digital light units per square millimeter (DLU/mm^2^). Because ROI measurements represented technical replicates derived from a limited number of biological samples, autoradiography data were analyzed descriptively (mean ± SD), and no statistical hypothesis testing was performed.

Saturation binding assays: The saturation binding autoradiography protocol was adapted and optimized from our previously published method [50]. Thaw-mounted whole-brain sections (20 µM thickness) from female Sprague Dawley rats were pre-incubated in 50 mM Tris-HCl buffer (pH 7.4) for 30 min at room temperature. Sections were then incubated for 10 min with increasing concentrations of [^11^C]suzetrigine, ranging from 0 to 1.5 nM (0.05, 0.1, 0.3, 0.5, and 1.1 nM), to generate a binding saturation curve. To determine nonspecific binding, a parallel series of sections was pre-incubated with 70 nM unlabeled suzetrigine (about 100-fold higher than the reported IC_50_) for 10 min prior to radioligand incubation. After incubation, all sections were washed twice with fresh Tris-HCl buffer according to the standard autoradiography protocol as described above and then air-dried. Autoradiographic images were acquired using phosphor imaging plates and quantified with ImageJ v1.54 using the ROI extraction method described above. Radioligand concentrations were calculated from the known radioactivity (MBq) using calibration curves and the molar activity (MBq/nmol). Tissue weight was recorded, and total, nonspecific, and specific binding (fmol/mg tissue) versus radioligand concentration (nM, converted from activity using molar activity), and data were analyzed by nonlinear regression using GraphPad Prism v10.4.1 to estimate equilibrium dissociation constant (K_D_) and maximal binding capacity (B_max_).

### 3.4. In Vivo PET Imaging

Rodent PET acquisition: All animal procedures were reviewed and approved by the Institutional Animal Care and Use Committee (IACUC) of Massachusetts General Hospital, in accordance with the National Institutes of Health guidelines for the care and use of laboratory animals. Female Sprague Dawley rats (4–6 weeks old) were anesthetized with inhaled isoflurane (3% for induction, 1.5% for maintenance) in 100% oxygen carrier gas. Pretreatment solutions of suzetrigine (1 mg/kg), verapamil (1 mg/kg, P-gp-inhibitor), and A-803467 (1 mg/kg, selective Na_V_1.8 inhibitor) were prepared for intravenous administration by dissolving each compound in a vehicle consisting of 10% DMSO, 10% Tween-80, and 80% sterile saline to yield a final concentration of 1.0 mg/mL. Animals were assigned either to baseline (no pretreatment) or pretreatment groups, receiving the designated compound intravenously 10 min prior to tracer injection. Up to four rats were scanned simultaneously in a MultiScan™ LFER150 PET/CT scanner (Mediso, Budapest, Hungary). An anatomical CT was acquired for attenuation correction and image co-registration. Each rat received an intravenous injection of [^11^C]suzetrigine (25.9–33.3 MBq), and dynamic PET acquisition was initiated immediately upon tracer administration for a total of 90 min. Data were reconstructed into 29 frames with the following timing scheme: 8 × 15 s, 6 × 30 s, 4 × 60 s, 3 × 120 s, 3 × 300 s, 3 × 600 s, and 2 × 900 s.

Rodent PET image analysis: Attenuation and scatter corrections were performed using a CT-derived material map, and PET data were reconstructed with an ordered-subset expectation maximization (OSEM) algorithm (Nucline, Mediso Ltd.) with 10 iterations and 3 subsets. The reconstructed PET and CT images (DICOM format) were further processed using PMOD v3.907 (PMOD Technologies, Ltd., Zürich, Switzerland). Anatomical CT images, acquired in the same coordinate space as PET data, were automatically co-registered to a standard rat brain atlas (Schiffer rat atlas VOI set) using a normalized mutual information algorithm [51,52]. The resulting transformation matrix was applied to dynamic PET frames, and regional time–activity curves (TACs) were extracted using predefined atlas-based regions of interest (ROIs). TACs were normalized to SUV (g/mL) using subject body weight and injected dose. For the area under the TAC analysis, brain regional AUCs (0–90 min) were normalized to each subject’s whole-brain AUC to account for interindividual differences and potential P-g-mediated influences between baseline and suzetrigine pretreatment conditions. For image display, representative summed PET images (60–90 min) were averaged over time, masked to remove extracerebral signals, and smoothed with a Gaussian kernel matching the intrinsic PET scanner resolution [53].

## 4. Conclusions

This study represents the first attempt to develop and evaluate a PET tracer targeting the Na_V_1.8 in the CNS. We selected suzetrigine, a recently FDA-approved, non-opioid Na_V_1.8 inhibitor with established safety and subnanomolar potency, as a candidate scaffold. The compound was successfully radiolabeled with carbon-11 via *O*-methylation without altering its pharmacophore, yielding [^11^C]suzetrigine with high radiochemical purity and molar activity. In silico docking confirmed stable interactions with the A-803467 binding pocket of human Na_V_1.8, and CNS MPO analysis indicated borderline yet plausible brain penetrability. In vitro autoradiography demonstrated saturable, selective binding of [^11^C]suzetrigine in rat brain sections with an apparent target density and high binding affinity, confirming sufficient binding site density for potential in vivo imaging.

Dynamic PET imaging in rats revealed rapid brain entry of [^11^C]suzetrigine but atypical tracer behavior under pharmacological pretreatments. Pretreatment with unlabeled suzetrigine or the P-gp inhibitor verapamil both produced marked increases in brain uptake, consistent with a strong interaction between [^11^C]suzetrigine and P-gp at the BBB. This interaction likely limits baseline brain penetration and hinders quantitative assessment of Na_V_1.8 binding in vivo. The modest uptake increase observed after pretreatment with the heterologous inhibitor A-803467 may instead reflect peripheral target occupancy or altered tracer free fraction rather than displacement of central binding sites. Because a validated reference region or arterial input function was not available, kinetic modeling could not be performed; therefore, the current findings should be interpreted qualitatively as feasibility data. Although the TAC profiles, characterized by rapid peak uptake followed by monotonic washout, did not exhibit the delayed accumulation typically associated with brain-penetrant radiometabolites, the possibility that early formed metabolites contributed to the PET signal, particularly under P-gp inhibition, cannot be excluded. Future studies incorporating arterial sampling and full radiometabolite characterization will be required to distinguish parent tracer kinetics from potential metabolites.

Future studies will focus on reducing P-gp interaction and improving CNS delivery, including through in vitro MDR1 efflux assays, plasma-protein binding measurements, and rational refinement of the tracer scaffold. Imaging under constant P-gp inhibition or in higher species such as non-human primates, together with arterial blood sampling for radiometabolite analysis and full kinetic modeling, will enable more definitive characterization of tracer pharmacokinetics and target specificity. Importantly, P-gp activity is often diminished in pathological conditions such as inflammatory or neuropathic pain, suggesting that altered tracer retention may offer a dual opportunity: to visualize Na_V_1.8 expression and to interrogate BBB function in disease. While our saturation binding studies show that Na_V_1.8 binding sites are present in rat brain tissue at levels compatible with PET detectability, protein expression may differ across species. Therefore, quantifying Na_V_1.8 binding site density in non-human primate and human brain tissues will be essential to guide future translational imaging efforts.

Overall, this proof-of-concept work highlights both the promise and the challenges of developing CNS-penetrant Na_V_1.8 PET tracers, emphasizing the critical influence of efflux transporters on ion-channel imaging probe design.

## Data Availability

The original contributions presented in the study are included in the article and Supplementary Materials. Further inquiries can be directed to the corresponding author.

## Supplementary Materials

The following supporting information can be downloaded at: https://www.mdpi.com/article/10.3390/ph18121816/s1, Table S1: Experimental details of [^11^C]suzetrigine radiosynthesis (*n* = 3); Figure S1: Semi-preparative HPLC purification of [^11^C]suzetrigine ([^11^C]**2**); Figure S2: Analytical HPLC of isolated [^11^C]**2**; Figure S3: Analytical HPLC characterization of [^11^C]**2** with the co-injection of the reference standard (**2**); Characterization of desmethyl precursor (**1**) and suzetrigine (**2**) (^1^H- and ^13^C-NMR spectra); Figure S4: 2D ligand–protein interaction maps for suzetrigine and A-803467 in Na_V_1.8 (PDB 7WE4); Table S2: Six descriptors for CNS-MPO scores of suzetrigine; Figure S5: In vitro autoradiography saturation binding with 70 nM unlabeled suzetrigine; Figure S6: In vitro autoradiography of [^11^C]suzetrigine binding in rat brain sections with co-incubation of verapamil; Figure S7: Regional time–activity curves (TACs) of [^11^C]suzetrigine in putative Na_V_1.8 enriched regions under baseline and homologous pretreatment conditions.

## Abbreviations

AUC: area under curve; BBB: blood–brain barrier; B_max_: total number of available binding sites (target protein density); ClogP: calculated partition coefficient; ClogD: calculated distribution coefficient; CNS: central nervous system; CT: computed tomography; DMF: dimethylformamide; DMSO: dimethyl sulfoxide; DLUs: digital light units; DRG: dorsal root ganglion; EOS: end of synthesis; FDA: Food and Drug Administration; HBDs: hydrogen-bond donors; HPLC: high-performance liquid chromatography; IACUC: Institutional Animal Care and Use Committee; IFD: Induced Fit Docking; I.V.: intravenous; K_D_: equilibrium binding constant; MPO: multiparameter Optimization; MW: molecular weight; Na_V_1.8: voltage-gated sodium channel subtype 1.8; NSAIDs: non-steroidal anti-inflammatory drugs; PET: positron emission tomography; PDB: Protein Data Bank; P-gp: P-glycoprotein; pK_a_: ionization constant; ROI: region of interest; SPE: solid-phase extraction; SD: standard deviation; SUV: standardized uptake value; TAC: time–activity curve; TBAOH: tetrabutylammonium hydroxide; TFA: trifluoroacetic acid; TPSA: topological polar surface area; TSPO: translocator Protein; 2D: two-dimensional; 3D-MLEM: three-dimensional maximum likelihood expectation maximization.
