# Supplementary material for: Toward Brain NaV1.8 Imaging with [11C]Suzetrigine
Source: Pharmaceuticals (Basel). 2025 Nov 28;18(12):1816. doi: 10.3390/ph18121816 (PMC12735773; doi:10.3390/ph18121816)
Supplement: Supplementary file 1 [file pharmaceuticals-18-01816-s001.zip › pharmaceuticals-4002339-supplementary.pdf]

Supplementary Material

Toward Brain $Na_V1.8$ Imaging with [^{11}C]Suzetrigine

Ramya Tokala[1], Torben D. Pearson[1], Braeden A. Mair[1], Sarah Bricault[1], Rachel Wallace[1], Hsiao-Ying Wey[1], Jacob M. Hooker[1], So Jeong Lee[1,*]

[1] Athinoula A. Martinos Center for Biomedical Imaging, Department of Radiology, Massachusetts General Hospital, Harvard Medical School, Charlestown, Massachusetts 02129, United States.

* Correspondence: slee178@mgh.harvard.edu

Contents

1. Radiochemistry……………………………………………………………………………………..……S2
   a. Materials and equipment…………………………………………………………….……………...……..S2
   b. Preparation of [^{11}C]methyl iodide……………………………………………………..……………..……S2
2. HPLC analyses…………………………………………………………………………………………..…S3
3. Characterization of suzetrigine and desmethyl precursor..…………...……………………….……………S4
4. 2D ligand-protein interaction maps for suzetrigine and A-803467 in $Na_V1.8$ (PDB: 7WE4)……………..S7
5. *In silico* CNS-MPO prediction…………………………………………………………………………..S8
6. *In vitro* autoradiography saturation binding with 70 nM unlabeled suzetrigine……………………………S8
7. *In vitro* autoradiography competition study with the P-gp inhibitor verapamil……………………………S9
8. *In vivo* regional brain time-activity curves………………………………………………………………...S10

1. Radiochemistry

Materials and equipment

Both desmethyl precursor of suzetrigine (**1**, CAS Number.: 2649467-91-2) and suzetrigine (**2**, CAS Number.: 2649467-58-1) were commercially available and procured from WuXi AppTec Co., Ltd. The quality of the compounds was confirmed by NMR and Mass spectrometry (see **Characterization of suzetrigine and desmethyl precursor** section). All of the commercialized chemicals (N, N-Dimethyl formamide and TBAOH-Tetrabutylammonium hydroxide 1.0 M in Methanol) were limited to ACS grade, procured from Sigma Aldrich and used directly. Except as otherwise noted, all commercial reagents were used without further characterization. Semi-preparative purification was performed on an Agilent Technologies HPLC 1200 series equipped with degasser (Model No. G1322A), quaternary pump (Model No. G1311A) and interface (Model No. 35900e). Analytical separation was conducted on an Agilent Technologies HPLC 1200 series fitted with same series of degasser, quaternary pump, autosampler (Model No. G1329A), diode–array detector (Model No. G1315B), radio detector (Model No. G1369A).

Preparation of [^{11}C]methyl iodide

[^{11}C]CO_2 was obtained via the ^{14}N (p, α) ^{11}C reaction on nitrogen with 2.5% oxygen, with 11 MeV protons (Siemens Eclipse cyclotron, Siemens Healthcare GmbH, Erlangen, Germany), and trapped on molecular sieves in a TRACERlab FX-MeI synthesizer (General Electric, G.E. Healthcare, Boston, MA, USA). [^{11}C]CH_4 was obtained by the reduction of [^{11}C]CO_2 in the presence of Ni/hydrogen at 350 °C and recirculated through an oven containing I_2 to produce [^{11}C]CH_3I via a radical reaction.

Table S1. Experimental details of [^{11}C]suzetrigine ([^{11}C]**2**) radiosynthesis (n=3)

Experiment No.	Volume (mL)	Molar Activity* (ndc, MBq/nmol)	Yield* (ndc, %)	Yield* (dc, %)	Time (min)	RCP (%)
1	10	21.1	6.7	15.8	35	> 98
2	5	118.4	4.6	19.5	38	> 98
3	5	47.0	5.9	22.3	39	> 98

*calculated at the end of synthesis (EOS); ndc = non-decay corrected; dc = decay corrected; RCP = radiochemical purity

2. HPLC analysis

Semi-preparative purification

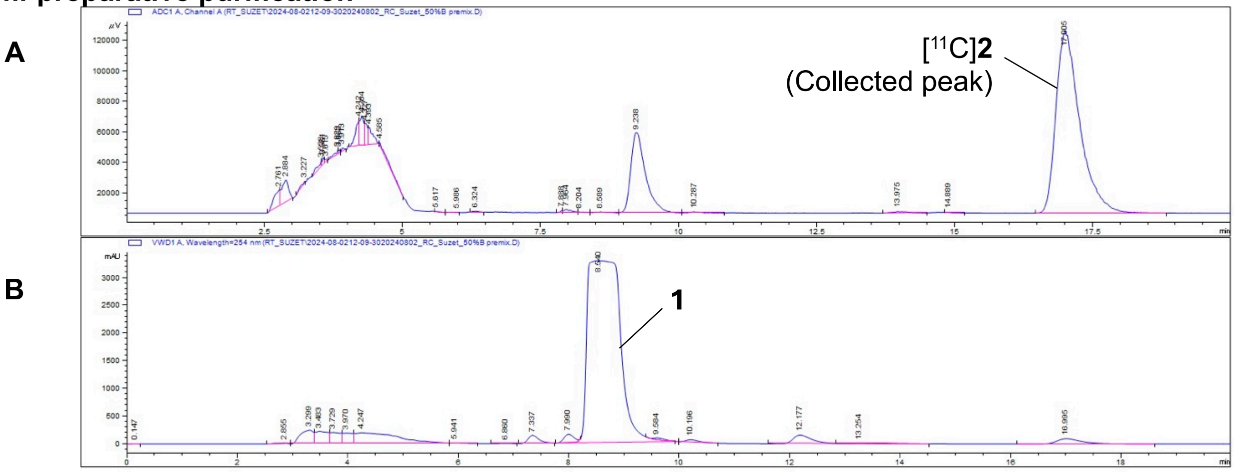

Figure S1. Semi-preparative HPLC purification of [11C]suzetrigine. (**A**) Radio-profile of [11C]methylation reaction mixture and the collected peak of [11C]suzetrigine ([11C]**2**, t_R = 17.00 min). (**B**) Simultaneous UV-profile showing separation of residual precursor (**1**) and other impurities.

Analytical HPLC

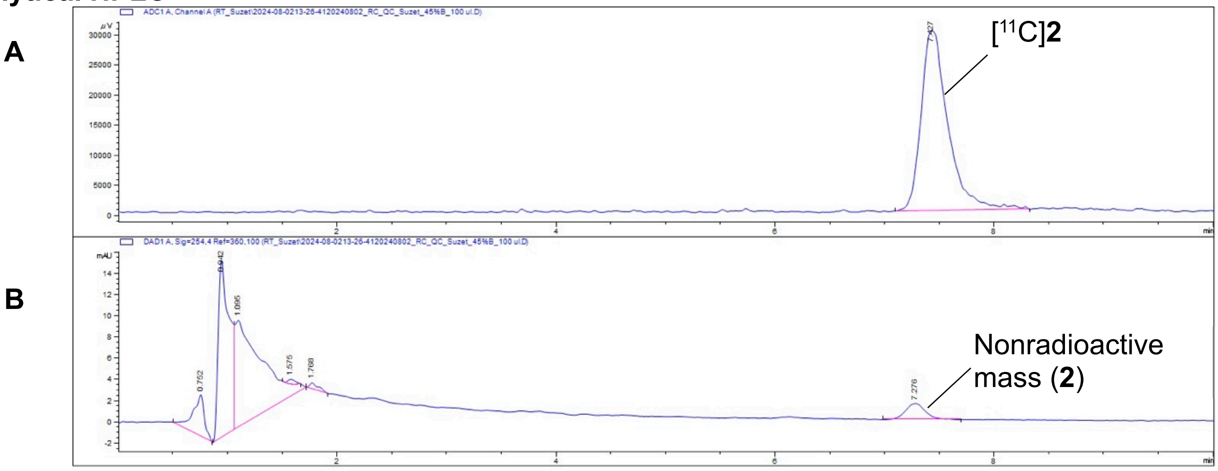

Figure S2. Analytical HPLC of isolated [11C]**2** peak from the semi-preparative HPLC. (**A**) Radio-profile: The peak at 7.42 min represents [11C]suzetrigine ([11C]**2)** showing > 98% radiochemical purity. (**B**) UV-profile: The corresponding UV signal of nonradioactive mass of **2.**

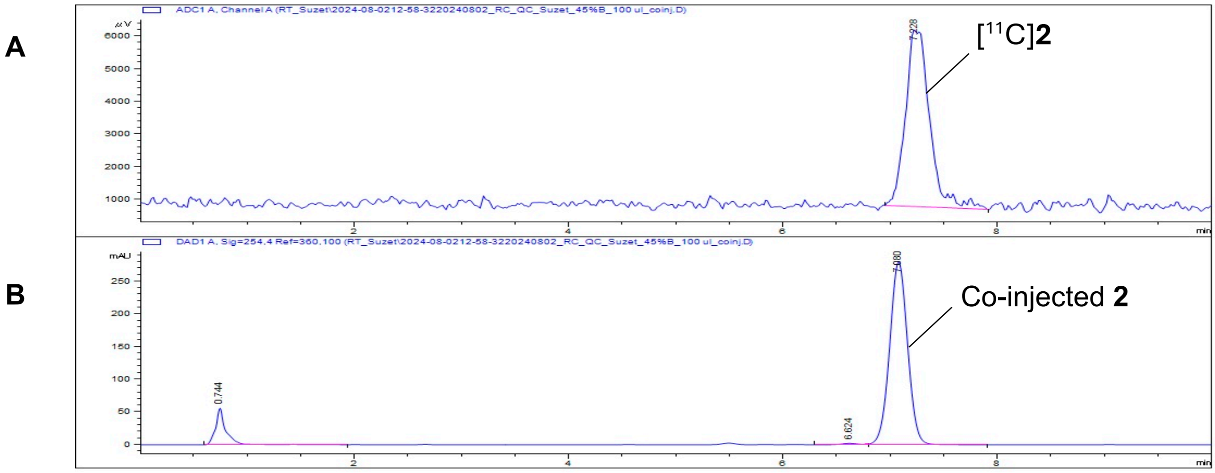

Figure S3. Analytical HPLC characterization of [11C]**2** with the co-injection of the reference standard (**2**). Radio-peak (A) corresponding to UV-peak (B) of 2 identify the collected C-11 peak as suzetrigine.

3. Characterization of suzetrigine and desmethyl precursor of suzetrigine

NMR spectra were obtained on a Bruker Avance HD 500 (500 MHz for ^{1}H; 125.77 MHz for ^{13}C) spectrometer. All ^{13}C NMR data presented are proton-decoupled ^{13}C NMR spectra, unless noted otherwise. ^{1}H and ^{13}C NMR chemical shifts (δ) are reported in parts per million (ppm) relative to TMS with the residual solvent peak used as an internal reference. ^{1}H NMR multiplicities are reported as follows: singlet (s), doublet (d), doublet of doublets (dd), doublet of doublets of doublets (ddd), quintet (q) and multiplet (m).

Desmethyl precursor of suzetrigine (1)

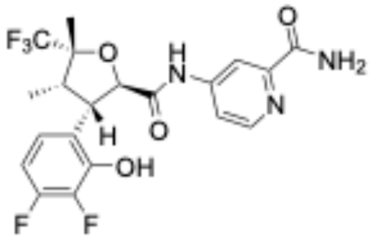

4-((2*R*,3*S*,4*S*,5*R*)-3-(3,4-difluoro-2-hydroxyphenyl)-4,5-dimethyl-5-(trifluoromethyl)tetrahydrofuran-2-carboxamido)picolinamide: ^{1}H NMR (500 MHz, DMSO-d_6): δ 10.72 (s, 1H), 10.46 (s, 1H), 8.45 (d, J = 5.5 Hz, 1H), 8.23 (d, J = 2.1 Hz, 1H), 8.05 (d, J = 2.8 Hz, 1H), 7.80 (dd, J = 5.5, 2.2 Hz, 1H), 7.60 (d, J = 2.8 Hz, 1H), 6.99 (ddd, J = 8.4, 5.8, 1.9 Hz, 1H), 6.87 – 6.78 (m, 1H), 5.06 (d, J = 10.2 Hz, 1H), 4.21 (dd, J = 10.2, 7.5 Hz, 1H), 2.80 (q, J = 7.5 Hz, 1H), 1.56 (s, 3H), 0.70 – 0.62 (m, 3H); ^{13}C NMR (125 MHz, DMSO-d_6) δ 169.7, 166.2, 151.9, 150.0, 146.6, 125.1, 123.3, 121.7, 116.1, 112.5, 107.0, 106.8, 86.0, 80.5, 45.2, 43.5, 23.4, 21.3, 14.6, 12.2; LCMS: *m/z* calculated for $C_{21}H_{20}F_5N_3O_4$ 460.1 found 460.1 $[M+H]^+$.

Suzetrigine (2)

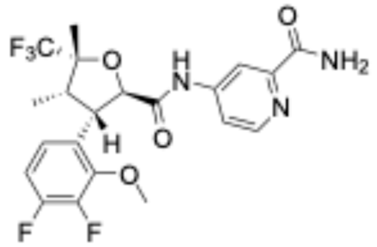

4-((2*R*,3*S*,4*S*,5*R*)-3-(3,4-difluoro-2-methoxyphenyl)-4,5-dimethyl-5-(trifluoromethyl)tetrahydrofuran-2-carboxamido)picolinamide: ^{1}H NMR (500 MHz, DMSO-d_6): δ 10.70 (s, 1H), 8.45 (d, J = 5.5 Hz, 1H), 8.24 (d, J = 2.2 Hz, 1H), 8.05 (d, J = 2.8 Hz, 1H), 7.79 (dd, J = 5.5, 2.2 Hz, 1H), 7.60 (d, J = 2.8 Hz, 1H), 7.20 – 7.05 (m, 2H), 5.07 (d, J = 10.2 Hz, 1H), 4.21 (dd, J = 10.2, 7.7 Hz, 1H), 3.91 (d, J = 2.2 Hz, 3H), 2.73 (q, J = 7.4 Hz, 1H), 1.57 (s, 3H), 0.69 (d, J = 4.5 Hz, 3H); ^{13}C NMR (125 MHz, DMSO-d_6): δ 169.5, 166.2, 151.9, 150.0, 146.6, 142.9, 127.4, 125.9, 125.1, 123.9, 116.2, 112.5, 111.4, 111.3, 86.0, 80.6, 62.1, 45.0, 44.2, 23.4, 12.3; LCMS: *m/z* calculated for $C_{21}H_{20}F_5N_3O_4$ 474.1 found 474.2 $[M+H]^+$.

NMR Spectra

Desmethyl precursor of suzetrigine (1)

^{1}H NMR spectra

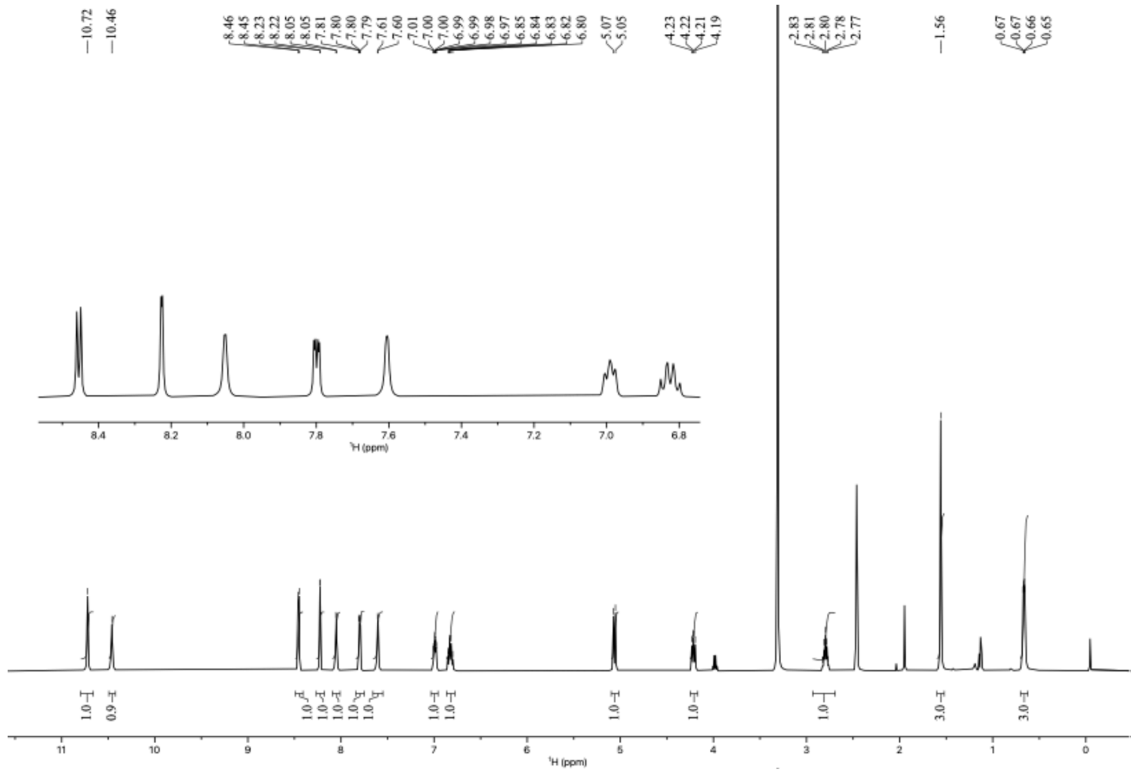

^{13}C NMR spectra

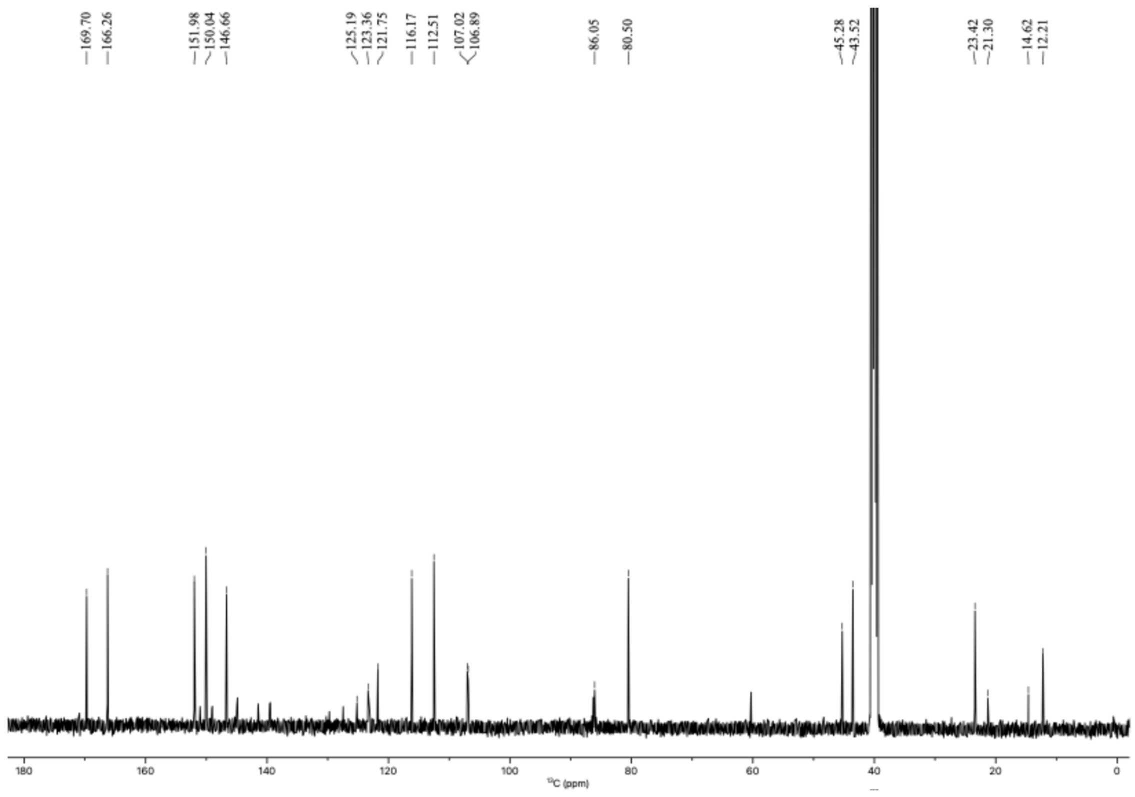

Suzetrigine (2)

^{1}H NMR spectra

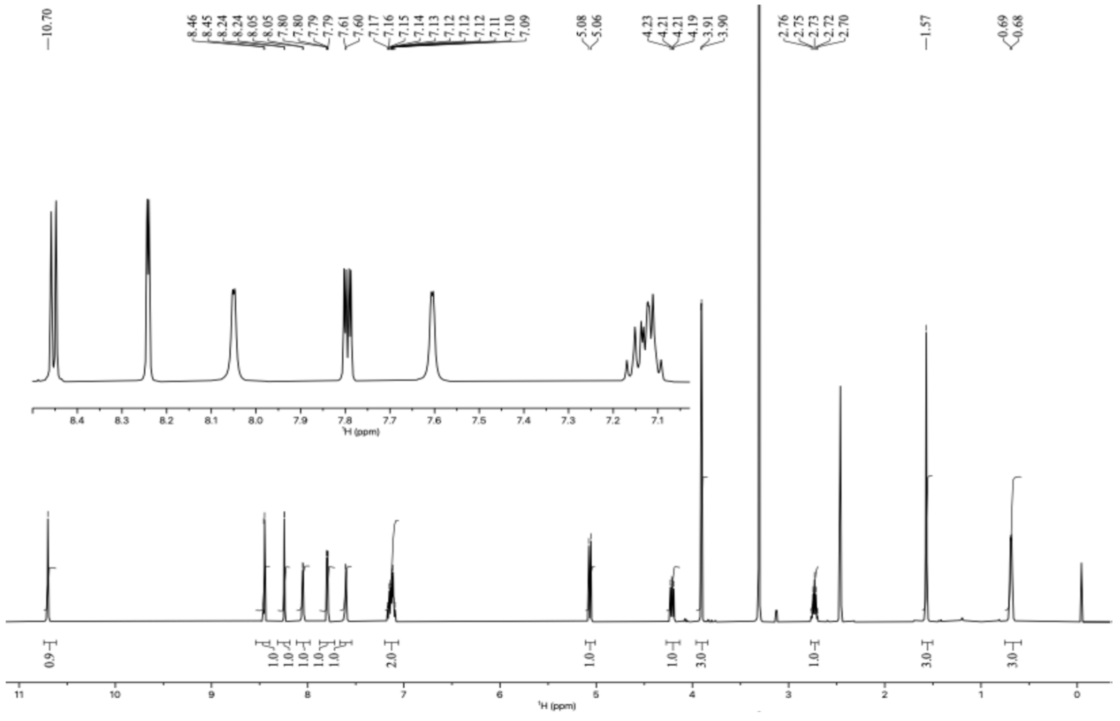

^{13}C NMR spectra

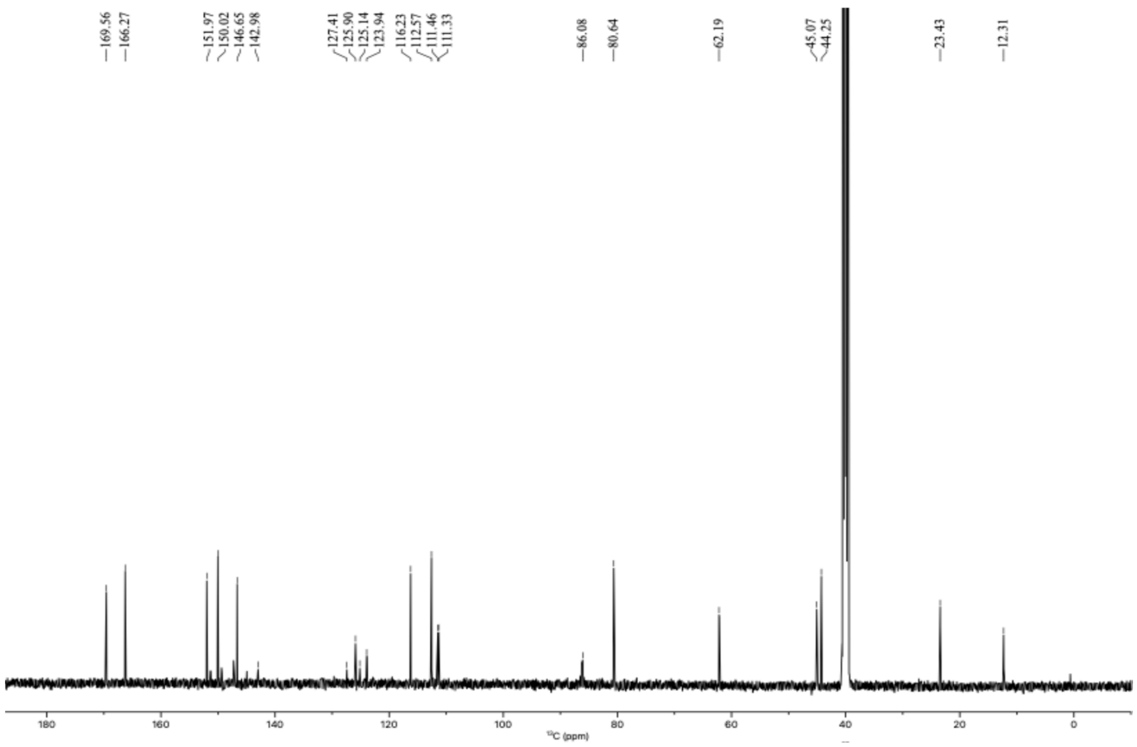

4. 2D ligand-protein interaction maps for suzetrigine and A-803467 in Na$_V$1.8 (PDB: 7WE4)

(A) Suzetrigine

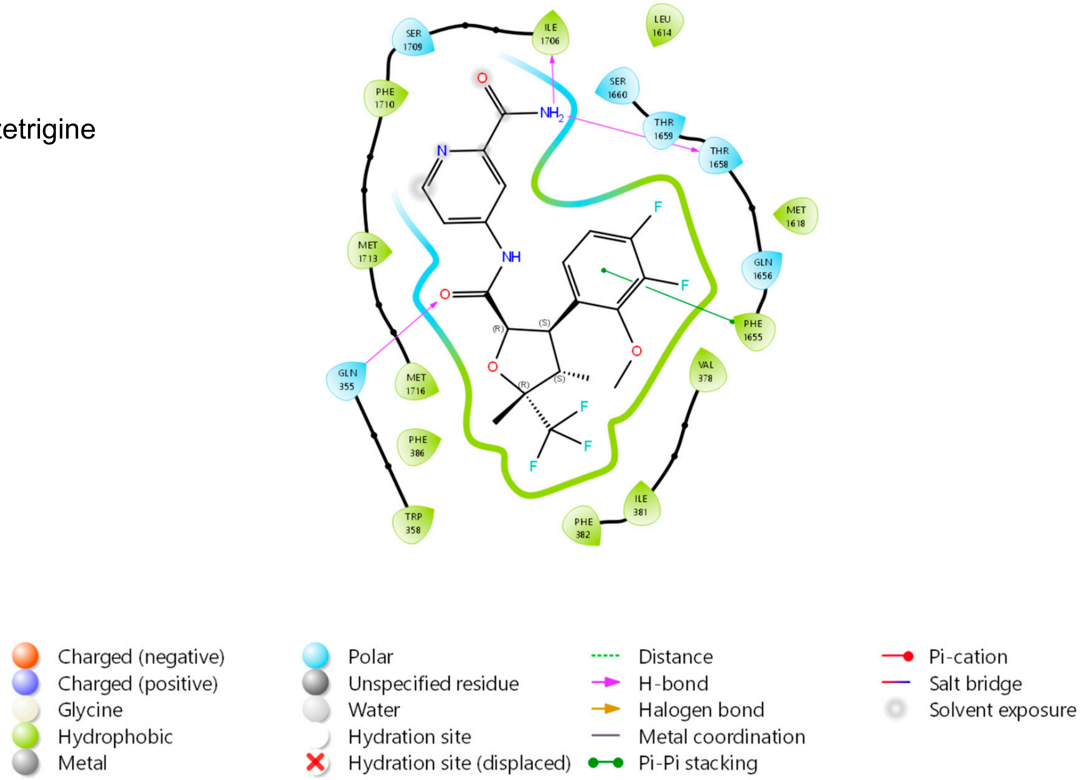

(B) A-803467

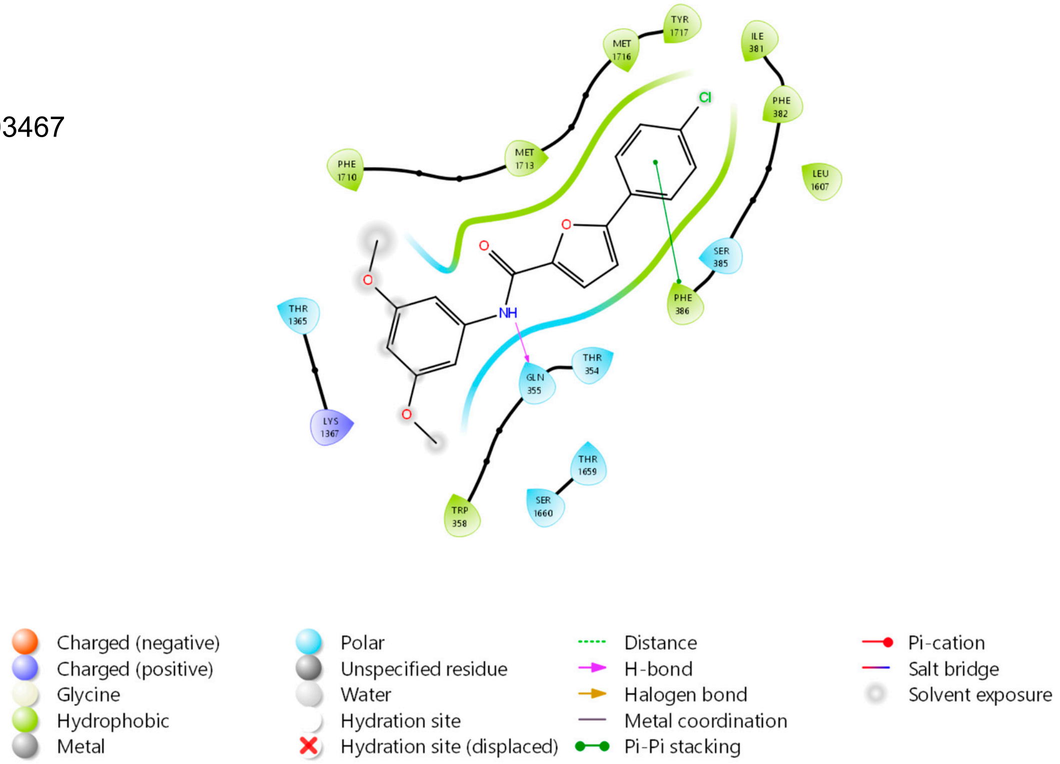

Figure S4. Two-dimensional (2D) interaction maps of suzetrigine and A-803467 within the Na$_V$1.8 binding pocket (PDB: 7WE4). (A) 2D ligand-protein interaction profile of suzetrigine, illustrating hydrogen bonds, π-π stacking, hydrophobic, and polar interactions with amino acid residues in the Na$_V$1.8 active site. (B) Corresponding interaction profile for the co-crystallized ligand A-803467, showing its contacts within the same binding pocket. These maps highlight the shared and distinct interaction features that support both ligands engaging the identical Na$_V$1.8 binding site.

5. *In silico* CNS-MPO prediction

Entry No.	Property	Value	CNS-MPO
1	ClogP	2.96	1.00
2	ClogD	2.96	0.52
3	MW	473.40	0.19
4	TPSA	103.54	0.55
5	HBD	3.00	0.25
6	pK_a	2.00	1.00
	TOTAL		***3.51***

Table S2. Six descriptors for CNS-MPO scores of suzetrigine

6. *In vitro* autoradiography saturation binding with 70 nM unlabeled suzetrigine

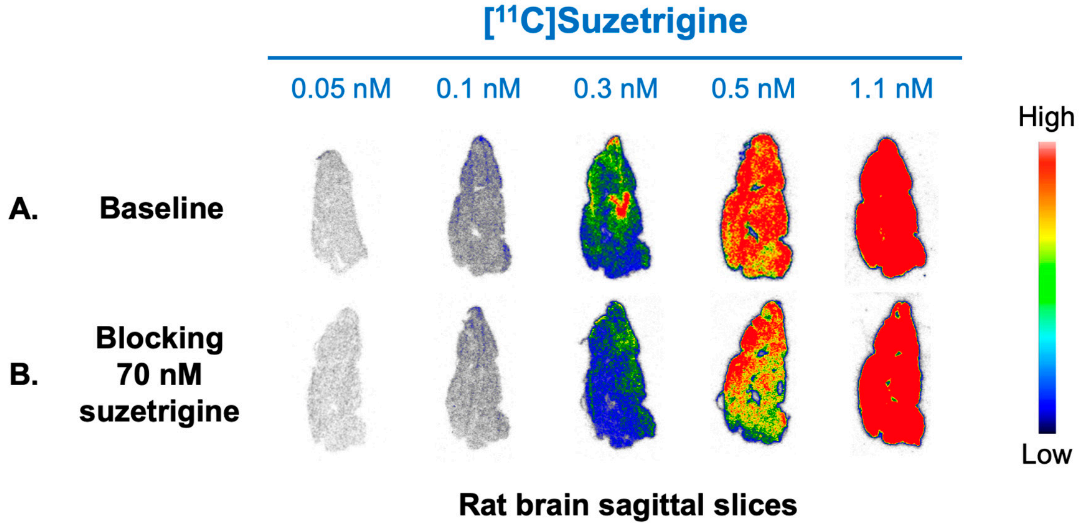

Figure S5. Representative autoradiography images showing total and non-specific binding following incubation with increasing concentrations of [^{11}C]suzetrigine (0.05, 0.1, 0.3, 0.5, and 1.1 nM) and treatment with a fixed blocking concentration (70 nM) of unlabeled suzetrigine. (A) Baseline: total binding of [^{11}C]suzetrigine. (B) Blocking with 70 nM suzetrigine: non-specific binding of [^{11}C]suzetrigine.

7. *In vitro* autoradiography competition study with the P-gp inhibitor verapamil

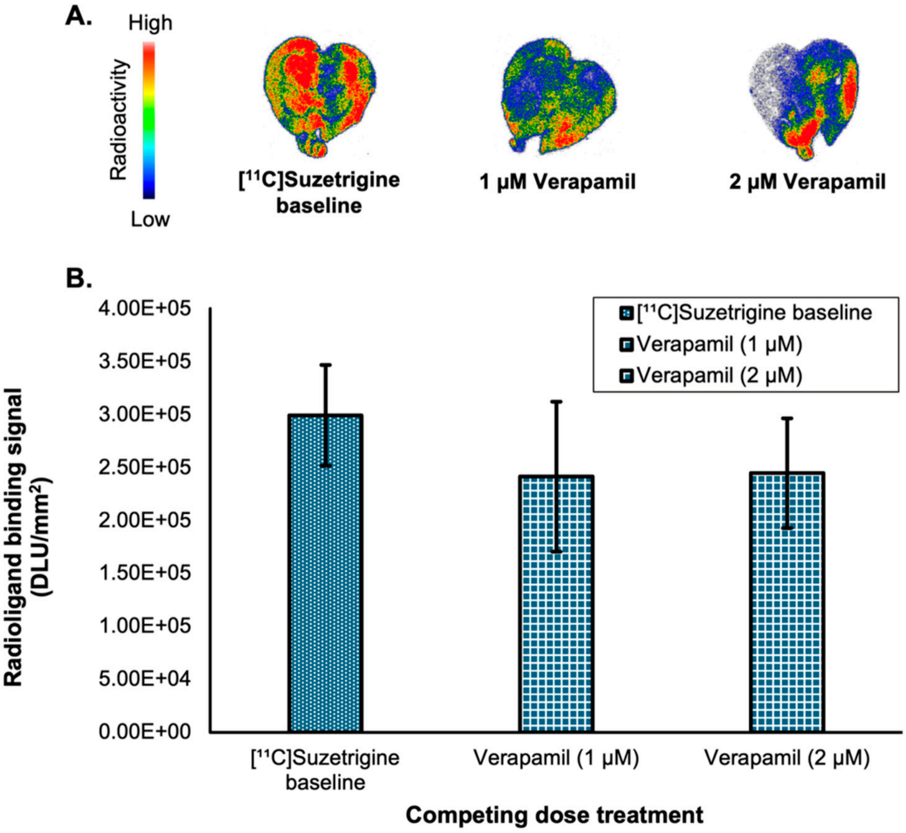

Figure S6. *In vitro* autoradiography of [^{11}C]suzetrigine binding in rat brain sections with co-incubation of verapamil. (A) Representative autoradiography image showing radio-signal uptake under [^{11}C]suzetrigine baseline and that with co-incubation of verapamil (1 and 2 µM). (B) Quantification of radiotracer uptake is presented as mean ± standard deviation. Unbiasedly selected image-based regions of interest (ROIs) were analyzed with sample sizes of n=8 for baseline and verapamil (1 µM); n=4 for verapamil (2 µM).

8. *In vivo* regional brain time-activity curves

A. *Unnormalized* regional TACs: [^{11}C]suzetrigine baseline vs. homologous pretreatment

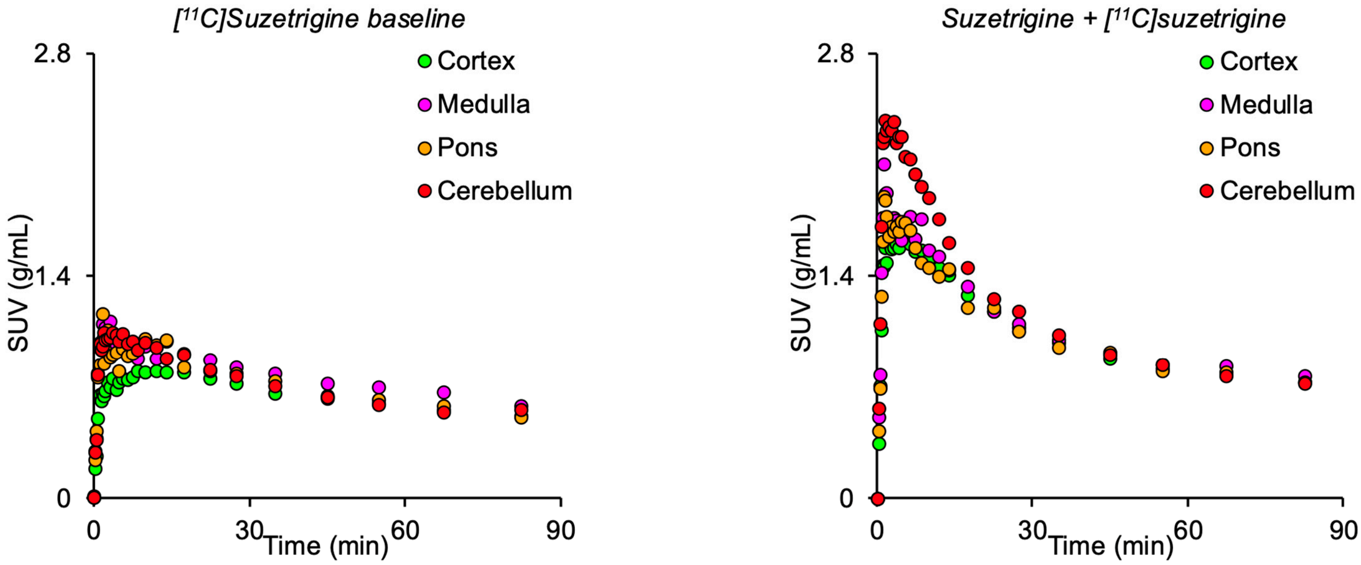

B. *Normalized* regional TACs to whole brain AUCs: [^{11}C]suzetrigine baseline vs. homologous pretreatment

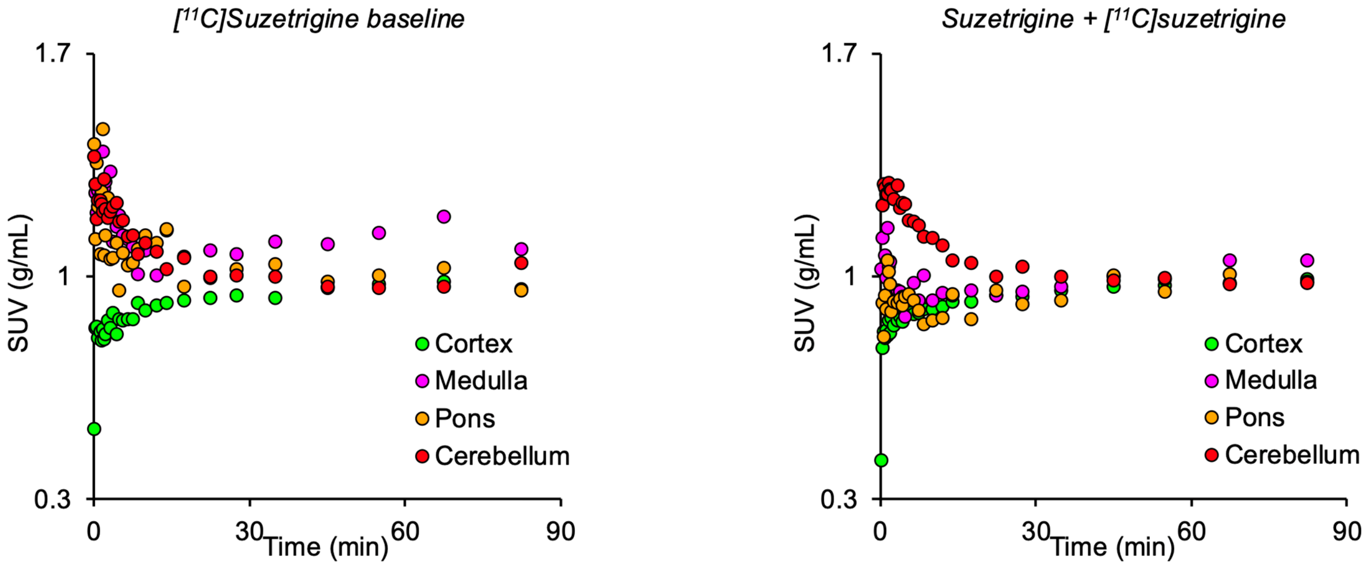

Figure S7. Regional time-activity curves (TACs) of [^{11}C]suzetrigine in putative Na$_V$1.8 enriched regions under baseline and homologous pretreatment conditions. (A) Unnormalized regional TACs comparing baseline ([^{11}C]suzetrigine) and homologous pretreatment (suzetrigine + [^{11}C]suzetrigine) scans. (B) Normalized regional TACs showing tracer uptake after adjustment to whole brain TAC. Each point in both (A) and (B) represents n=2.
